# Classifying Streamflow Duration: The Scientific Basis and an Operational Framework for Method Development

**DOI:** 10.3390/w12092545

**Published:** 2020-09-11

**Authors:** Ken M. Fritz, Tracie-Lynn Nadeau, Julia E. Kelso, Whitney S. Beck, Raphael D. Mazor, Rachel A. Harrington, Brian J. Topping

**Affiliations:** 1 Center for Environmental Measurement and Modeling, Office of Research and Development, US Environmental Protection Agency, Cincinnati, OH 45268, USA; 2 Region 10, US Environmental Protection Agency, Portland, OR 97205, USA; 3 Office of Wetlands, Oceans, and Watersheds, US Environmental Protection Agency, Washington, DC 20460, USA; 4 Oak Ridge Institute for Science and Education Fellow, Oak Ridge, TN 37831, USA; 5 Southern California Coastal Water Research Project, Costa Mesa, CA 92626, USA

**Keywords:** classification, flow duration, indicators, perennial, intermittent, ephemeral, temporary, flow permanence, intermittency, rapid assessment

## Abstract

Streamflow duration is used to differentiate reaches into discrete classes (e.g., perennial, intermittent, and ephemeral) for water resource management. Because the depiction of the extent and flow duration of streams via existing maps, remote sensing, and gauging is constrained, field-based tools are needed for use by practitioners and to validate hydrography and modeling advances. Streamflow Duration Assessment Methods (SDAMs) are rapid, reach-scale indices or models that use physical and biological indicators to predict flow duration class. We review the scientific basis for indicators and present conceptual and operational frameworks for SDAM development. Indicators can be responses to or controls of flow duration. Aquatic and terrestrial responses can be integrated into SDAMs, reflecting concurrent increases and decreases along the flow duration gradient. The conceptual framework for data-driven SDAM development shows interrelationships among the key components: study reaches, hydrologic data, and indicators. We present a generalized operational framework for SDAM development that integrates the data-driven components through five process steps: preparation, data collection, data analysis, evaluation, and implementation. We highlight priorities for the advancement of SDAMs, including expansion of gauging of nonperennial reaches, use of citizen science data, adjusting for stressor gradients, and statistical and monitoring advances to improve indicator effectiveness.

## 1. Introduction

Because streamflow influences patterns and processes in streams and adjacent riparian areas, streamflow classification is often used to support environmental management and restoration decisions. While gauging stations provide direct hydrological data for streamflow classification, the cost of maintaining gauging networks limits their lifespan and geographical extent [1]. For instance, gauges tend to be located on large, generally perennially flowing streams and rivers, so most of the stream miles in the United States (U.S.) are underrepresented by the gauge network [2]. Being less expensive, the deployment of data loggers [3,4] can fill gaps missed by gauge networks, but these approaches still require substantial effort and have a time lag in supplying data for classification. The National Hydrographic Dataset (NHD) is the most comprehensive source on stream extent and streamflow classification in the U.S.; however, the NHD is a static characterization that also tends to more accurately characterize larger streams and rivers than the more abundant headwater streams [5,6]. Unlike gauges, remote sensing approaches have the potential to characterize hydrology across landscapes [7,8]. Nonetheless, the coarse temporal resolution and constrained ability to differentiate water surface from the bed surface in shallow flowing water in networks through dense tree canopies are limitations of current remote sensing technology to inform comprehensive streamflow classification. Consequently, reliable hydrography information (i.e., channel extent, flow duration class) is not always available where needed to support management decisions.

Streamflow duration indicators are rapid, cost-effective alternatives to gauging and remote sensing that provide reach-specific classifications. Our objective is to provide the scientific basis and an operational framework to use environmental indicators for streamflow duration classification at the reach scale (10^1^–10^2^ m). First, we describe streamflow duration classification and provide an overview of environmental indicators that can be used to infer flow duration because they serve as measures of either responses to, or controls of, flow duration. Next, we provide both conceptual and operational frameworks for the development of Streamflow Duration Assessment Methods (SDAMs), which are rapid assessments that use indicators to classify streamflow duration at the reach-scale. An SDAM includes the protocols for measuring indicators and an index or model to predict or draw a conclusion regarding the streamflow duration class of the targeted reach. We highlight the primary components and process steps needed for SDAM development, as well as alternative approaches, and key considerations. We close by highlighting future advances to further improve SDAMs and their application.

## 2. Streamflow Duration

**Figure 1.**
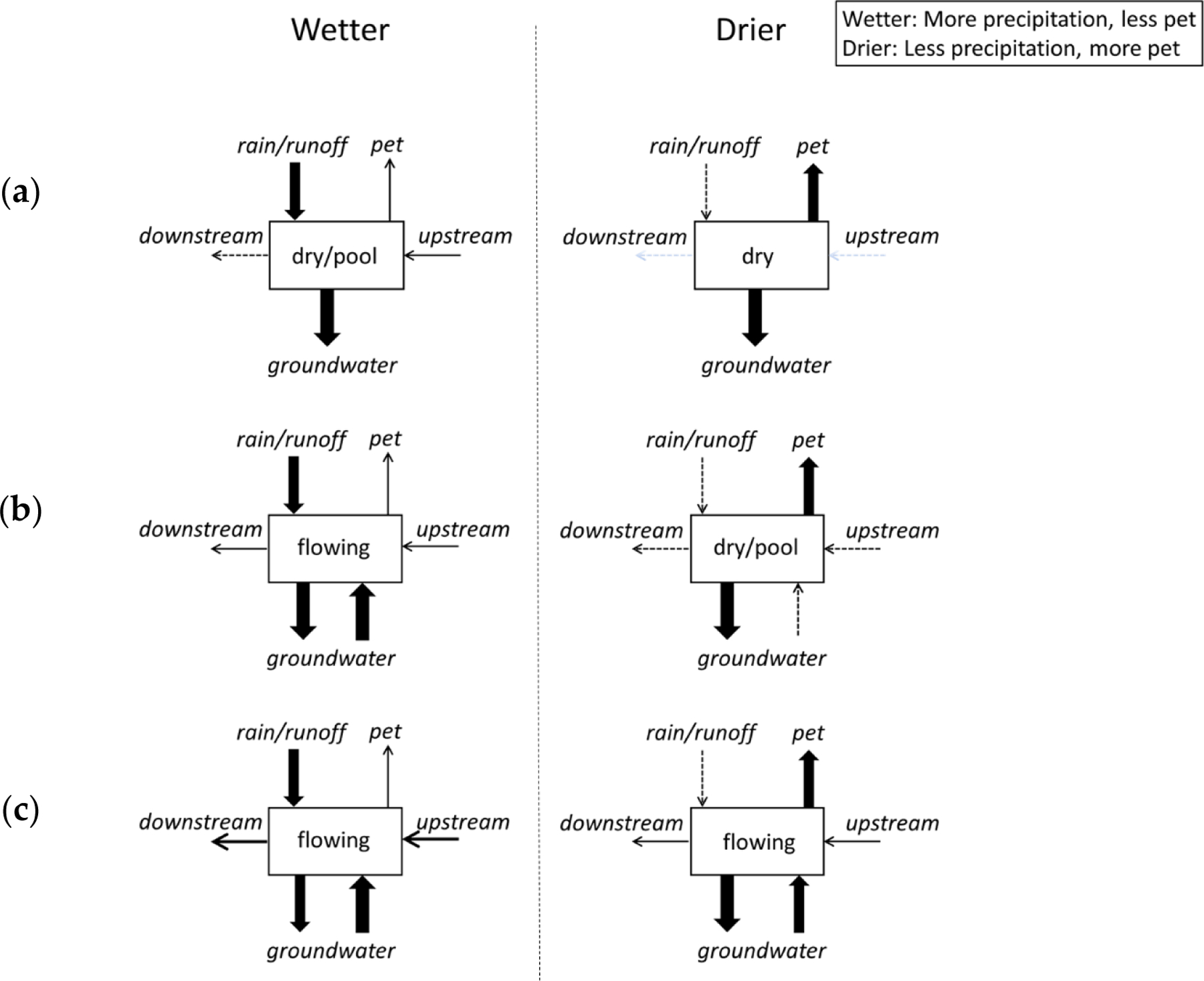
Conceptual figure illustrating differences among ephemeral (**a**), intermittent (**b**), and perennial (**c**) reaches during wetter and drier periods. Typical baseflow hydrologic conditions of the stream reach (box) are shown. The arrow thickness illustrates the relative magnitude of catchment inputs (rain/runoff, groundwater) and outputs (potential evapotranspiration (pet), groundwater) that influence hydrologic connection within a stream reach.

The presence of surface flow is a fundamental basis for stream classification. The presence and absence of surface flow represents a break along a gradient of hydrologic conditions (Figure 1). Surface flow can range from interstitial flow, where surface flow is visibly limited to flow between stones or organic material in shallow habitats or visible only at the tails and heads of pools; to overbank flooding, where the entire channel and adjacent floodplain may be submerged under flowing water [9,10]. The lack of surface flow can be reflected in one or more of the following conditions: pools of standing water; moist streambed sediment; completely dry sediment [11].

Classification of flow presence has historically focused on the temporal dimension, specifically on flow duration or continuity of flow through time. Perennial describes channel lengths having continuous surface flow that do not experience drying outside of extreme drought [9,12]. In contrast, channels that experience recurrent loss of surface flow are called nonperennial [13]. These can be further subdivided into intermittent and ephemeral. Intermittent channels are typically defined as having continuous surface flow for part of the year that is sustained by snowmelt and/or groundwater. In contrast, ephemeral channels are typically defined as flowing only during and immediately following precipitation or snowmelt [9,12]. Although there is not a universally accepted duration that separates intermittent and ephemeral flow, the timing of streamflow becomes more predictable going from ephemeral to intermittent to perennial within a given geographic area. Similarly, while there is not a universally accepted length of record needed to classify streamflow duration, it is generally accepted that streamflow duration classes represent the typical regime at a reach over many years. While a reach may change streamflow duration classes in the long term due to any number of factors (e.g., long-term water withdrawal or augmentation, channel headcutting, changing climatic conditions), the streamflow duration classification of a reach represents the typical regime and, therefore, does not change year to year.

To appropriately apply such a classification scheme based on gauge, survey, or indicator data, it is important to understand what streamflow duration classifications do not represent. For example, these flow classes do not describe the magnitude of streamflow, either in terms of individual events or cumulatively over longer periods. Although groundwater commonly supplies more consistent base flow to perennial and intermittent reaches [14], streamflow duration classes are not exclusively defined by the sources of streamflow. Previous studies have used the classification terminology to describe the spatial dimension of surface flow or connectivity through space. For instance, intermittent has been used to describe channels with pools interspersed along an otherwise dry channel [15,16]. A mosaic of such conditions can occur across a stream network at a given point in time, and these patterns are spatially controlled by natural and anthropogenic factors [17,18]. Because of recurrent drying of varying frequency, duration, and predictability, nonperennial streams can be considered transitional, representing a continuum between strictly aquatic and strictly terrestrial habitats.

## 3. Scientific Basis of SDAM Indicators

Environmental indicators are measurable properties that provide inference regarding a more complex phenomenon of interest [19]. Such surrogate measures are used because direct measurement of flow over time is either too difficult or resource intensive. These indicators may be environmental characteristics that control or govern streamflow duration (e.g., catchment features or climate), as well as those that respond (e.g., presence of long-lived taxa that depend on year-round flow) (Table 1). We note that control and response indicators are not necessarily mutually exclusive. However, we provide examples of indicators that could fall within each category, and we discuss the mechanisms governing the relationship between indicators and hydrology, as well as factors that mediate those relationships.

**Figure 2.**
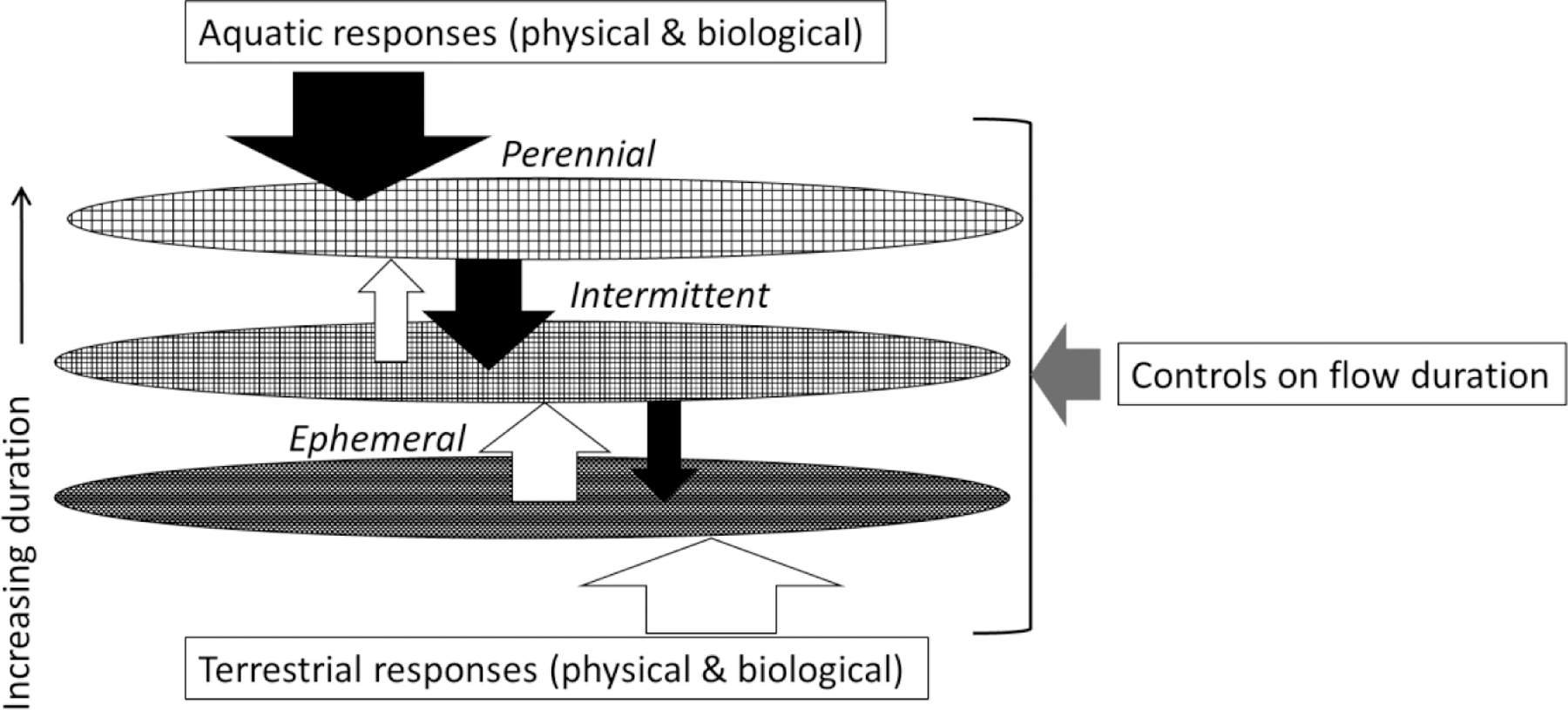
Conceptual figure illustrating indicators of streamflow duration. Response indicators are “filtered” along the gradient of streamflow duration, such that the strength of aquatic signal (black arrows; e.g., stream biota, fluvial geomorphology) declines and terrestrial signal (white arrows; e.g., terrestrial biota, soil development) increases with decreasing flow duration. Control indicators (gray arrow) are factors that govern streamflow duration from reach to landscape scales (e.g., meteorology, geology, land cover).

The environmental filter concept [42,43] portrays environmental conditions as selective filters on the distribution and abundance of species. Without the combination of traits (i.e., measurable properties of organisms that reflect behavior, life history, physio-morphological adaptation, resource use, and mobility) allowing persistence under certain environmental conditions, species are eliminated. We apply the environmental filter concept to illustrate streamflow duration classes as a series of designated filters along an environmental gradient with indicators represented as responses to, and controls of, flow duration (Figure 2). Therefore, our application also expands the environmental filter concept to include physical as well as biological responses and their relationships with control indicators acting on filters along the flow duration gradient. While our illustration of environmental filters represents perennial, intermittent, and ephemeral classes, a different number and positioning of filters along the flow duration gradient could be used to represent different flow duration classifications for SDAM development (see Table 2).

Aquatic and terrestrial responses are diametrically opposed along the gradient of flow duration, such that as the strength of one signal increases, the other decreases. Therefore, a suite of indicators that quantify aquatic and terrestrial responses to flow duration can be integrated into SDAMs and reflect the signals that concurrently increase and decrease along the flow duration gradient. Contraction from surface water-connected habitats to isolated pools, loss of all surface water, and drying of shallow subsurface sediments represents progressive reductions in the extent and variety of aquatic habitats available [56]. The loss of water volume can increase contaminant and pathogen concentrations and water temperatures and decrease dissolved oxygen [57–59]. Biological responses can, therefore, be in direct response to changes in habitat availability as well as changes in physicochemical quality and subsequent intensification of species interactions [60,61]. Conversely, inundation, groundwater accessibility, and continuous forces of flow associated with long streamflow duration limit edaphic processes and colonization of channel habitats by terrestrial biota [62–64].

Physical indicators, such as biological indicators, respond to hydrology. The physical environment of stream channels reflects the hydrology and transported sediment that shape channels and bed and bank material [65]. While periodic high magnitude flows are responsible for channel and floodplain maintenance (e.g., sediment transport, channel geometry, bed topography), it is the entire range of flows that shape the channel and floodplain environment [66]. The physical forms of stream channels are diverse, spanning a range within and across flow duration classes [67–69]. Flow duration, though an important gradient, is just one environmental dimension that contributes to channel form [70]. Sporadic flows (mostly large floods), downstream transmission losses, high sediment supplies and bedload, sparse vegetation, and easily erodible banks are commonly cited as causes for the predominant forms of ephemeral channels being braided channels and single-threaded planar-bed channels in arid regions [71]. Meandering channel morphology has been considered largely restricted to perennial rivers having riparian vegetation and cohesive bank material, but ephemeral meandering channels were widespread and had subtle differences from perennial meandering channels in arid regions [72]. The tightness of the bends (i.e., curvature ratio) and the meander wavelength (linear distance between bends) relative to the channel width in the ephemeral channels were, on average, lower than those of perennial channels [72]. Reid and Laronne [73] indicated that flow duration represents a gradient for describing bedload transport, whereby very high amounts of bedload are transported from ephemeral compared to perennial channels under the same flood magnitude. Limited opportunity for winnowing of fine sediments in ephemeral channels results in higher amounts of fine sediments within the upper layer of the streambed compared to the coarse armored surface layer of perennial channels. Hassan [30] differentiated gravel bars in ephemeral channels in Israel as being unsorted, intermixed with sand, and without downstream fining, whereas bars in perennial channels had armored surfaces and downstream fining. Physical indicators that differ among flow duration classes may reflect not just the difference in flow duration (or dry duration) but also other factors such as magnitude, timing, and frequency of flows within a given landscape.

Channel form reflects not only fluvial processes experienced during flow but also the counteracting terrestrial processes such as riparian vegetation growth, soil formation, and hillslope coupling [74]. As flow duration decreases, high magnitude flows become more infrequent, and the intervening time for terrestrial processes increases [75–77]. This is relevant to flow duration indicators because geomorphological characteristics associated with fluvial processes may be indicative of longer flow durations, whereas those associated with terrestrial processes may be indicative of shorter flow durations. However, the relative effect of fluvial versus terrestrial processes influencing channel form is complex and depends on factors such as climate, bed slope, and sediment supply that in turn influence the propensity for channel change by floods and subsequent recovery (or “healing”) [78]. For example, channels in arid regions are considered more prone to change because floods generally have higher magnitudes than in humid regions due to higher precipitation intensity and compact upland soils with little stabilizing vegetation and organic matter [79,80]; therefore, terrestrial and fluvial features may be evident at the same time in the same reach. The longitudinal position of reaches within a stream network also mediates the relative effect of fluvial versus terrestrial process on channel form. For example, in humid regions, headwater channels can have substantial structural complexity derived from terrestrial processes (e.g., large wood, tree roots, boulder, bedrock outcrops). Such structural complexity, even on steep terrain, is largely considered to be resistant to channel change from storm runoff as discharges are forced out of high roughness, shallow channels as overbank flow, interstitial flow, and interconnected overland flow [81], minimizing the influence of fluvial processes on channel form even where flow is perennial. In contrast, lower reaches in arid regions tend to have downstream flow volume decreases because of transmission losses, evapotranspiration, and lack of significant tributary inflows [70]. Thus, physical indicators reflecting fluvial or terrestrial processes should be evaluated in the context of other factors, such as physiography, longitudinal position, and adjacent land use.

In addition to properties representing responses to flow duration, SDAM indicators can include drivers of, or controls on, flow duration (Figure 2); these are properties that influence flow duration at the targeted reach. Surface flow in a reach occurs when upstream discharge is higher than the transport capacity of the underlying streambeds. Therefore, causes of drying are factors limiting water supply and/or channel transport capacity, including meteorology, geology, and land cover across a hierarchy of scales [17]. Meteorology includes water and energy input across increasing temporal scales from individual events (e.g., storm, cold front) to subannual weather patterns, multi-annual phenomena, and climate. Geology ranges from sediment grain size at the patch scale (e.g., 0.1–1 m) to subreach or habitat units (e.g., pools vs. riffles), individual reaches, and entire catchments. Land cover encompasses in-channel structures that govern flow, land-water interface along reaches, land uses within catchments, and subcontinental biomes. Here, we largely focus on catchment- to subreach-scale controls, whereas regional-scale controls are further discussed in relation to study reach selection.

Meteorology influences the proximity of the water table to the streambed elevation, the availability of water exceeding evapotranspiration and infiltration for streamflow, and when water is released from storage [17]. Surface flow duration within reaches represents a balance between supplied water and the extent and permeability of streambeds. Shallow water tables can supply flow to intersecting channels for varying lengths of time in response to recent precipitation. Shifts in the balance of evapotranspiration with water available for streamflow can occur at diel, seasonal, and multi-annual time scales [52,82,83]. Flow duration increases with more frequent, intense, and longer periods of precipitation. In addition to affecting contributions and deficits to flow, meteorology can affect flow duration through the flow timing and storage in areas draining snowpack and/or glaciers [84].

Geology describes conditions mediating how water is stored and transported through the river network. Characteristics such as catchment area, elevation, and topographic relief describe the position of reaches within catchments and provide information on the quantity, source, and storage capacity for water to reaches. Storage capacity and hydraulic conductivity are also key surficial geologic characteristics that influence flow duration. At the catchment scale, permeable surficial geology and soils enhance storage that can extend flow duration over dry periods [85]. Within reaches, however, thick layers of coarse unconsolidated bed sediments enhance transmissivity and subsurface transport capacity, diminishing surface flow duration [86,87], whereas thin bed sediment layers overlying impermeable layers can maintain surface flow for extended periods. Reach slope also affects flow duration; steeper topography will tend to have less permeable surface geology, and the water table will sit farther from the surface than in adjacent lowlands [88]. Flow duration can be higher in reaches with springs and seeps that emerge where preferential flow paths (fractures, contacts between contrasting geology, soil macropores) intersect channels and/or coincide with abrupt changes in slope, hydraulic properties, or high topographic convergence [89–91]. Because of local geological heterogeneity, flow duration may not reflect a gradual longitudinal gradient along streams (i.e., increasing or decreasing flow duration with catchment area) but a discontinuous one such that expansion-contraction dynamics reflect the coalescence and fragmentation of surface water connectivity [4,92].

Land cover describes the land surface conditions mediating how water is received and shunted, reflecting the interactions between meteorology, geology, and natural and human activities. For instance, catchment vegetation cover interacts with meteorology through changes in evapotranspiration. Increased impervious surface cover can lower flow duration by reducing recharge and water available for baseflow during dry periods, but year-round surface flows can be sustained by effluent discharges and/or elevated groundwater tables within incised channels [93,94]. Irrigation extraction and withdrawals can lower flow duration by diverting water that would otherwise flow within stream channels [95,96]. Riparian vegetation cover may be a response to flow duration but can be a control at the reach scale by affecting channel form, surface-subsurface exchange, and local groundwater levels [26,70,97]. Several studies have documented increases in streamflow following removal of riparian vegetation due to decreases in evapotranspiration [98,99]; however, riparian vegetation can sometimes increase streamflow when it plays a major role in intercepting fog that replenishes groundwater [100].

Physical and biological responses and/or controls of flow duration operate at a range of spatial and temporal scales (Table 1). Some indicators are meaningful at large scales but provide little discriminatory power at small scales or vice-versa. For instance, the heterogeneity of control or response variables is greater at large scales but is homogeneous over a smaller scale. Indicators across different scales may also interact with one another. For example, riparian plant assemblages of intermittent and perennial reaches were nested along an elevation gradient [101], showing that elevation mediates how riparian vegetation reflects flow duration class. Across forested headwater catchments in eastern U.S., ephemeral reaches had smaller catchment areas than intermittent and perennial reaches, but intermittent and perennial reaches differed by entrenchment ratio [36]. The discriminatory ability of some indicators to classify reaches by flow duration is affected by other environmental conditions linking flow duration to other gradients.

The discriminatory ability of indicators to classify reaches by flow duration can also be affected by other environmental conditions that vary over space and time. For example, response indicators may reflect not only flow duration, but also other aspects associated with source water or water quality. The distribution and cover of biota can be strongly linked to the physiochemistry of groundwater [102,103]. Disturbances are discrete events that disrupt natural systems and their components through exposure to stressors and can be broadly divided into those that are sustained (i.e., press) versus transient (i.e., pulse) in their temporal pattern [104]. Disturbances other than stream drying can reduce discriminatory ability of flow duration indicators if responses are indistinguishable or complex (e.g., synergistic, antagonistic; [105,106]. High impervious cover increases the frequency of moderate-sized stormflow events in arid streams [107,108], so differences among flow duration classes in sedimentary characteristics and channel form may be diminished. A major flood reduced the abundance and diversity of the macroinvertebrate assemblage to a greater extent at a downstream perennial reach than at upstream intermittent reaches in a tallgrass prairie stream network because flood magnitude downstream was ~30X greater than at intermittent reaches [109]. Indicator differences among flow classes will vary over time since disturbance. Progressive changes in the structure of biotic communities since rewetting [109,110] indicate that timing has a strong bearing on distinguishing intermittent from perennial and ephemeral reaches using biotic indicators. The time required for biotic assemblages to stabilize following disturbance is dependent upon biotic characteristics (e.g., growth rate, mobility, life history) and physical factors of the reach (e.g., refuge proximity and size, recent and historical flow regime). The time for physical characteristics to stabilize following disturbance is dependent upon climatic, geologic, and biological factors. For instance, higher magnitude floods can cause more enduring physical changes to channels in arid regions than in humid regions because the frequency of lower magnitude floods is lower, and the rate of terrestrial processes is much slower [74].

Because the goal of an SDAM is to classify reaches by flow duration, which represents the typical regime over many years, indicators reflecting flow duration over longer periods of time are favored over those that reflect more immediate flow conditions. While there are a variety of bed features (e.g., mud drapes, desiccation cracks, flaser bedding) that represent physical evidence of recent flow, their presence in a reach is typically transient and does not differentiate flow duration classes as these features can be left by flows of different magnitudes regardless of their duration [111]. Persistent fluvial features in channels are normally formed by high flows and, therefore, are more indicative of magnitude and frequency than flow duration alone. The persistence of geomorphological indicators depends upon the propensity for change and the capacity of the system to resist change [78]. Many physical features indicative of dry channels (e.g., aerially distributed leaf litter, evaporites, efflorescence, mud cracking) have low resistance to low and high magnitude flows and, therefore, are transient features. Although such indicators may be recurrent over space and time, their presence is likely to reflect recent or contemporary conditions. Many aquatic biota will not be evident when channels dry because they will disperse or have been consumed by scavengers [112,113]. However, some aquatic organisms have life stages that remain viable in dry streambeds (“seedbank” [114]). Others move to habitats within reaches that remain wetted, such as the hyporheic zone or isolated pools. Many taxa leave behind shells, cases, burrows, and exuviae as evidence of their recent presence [115–117]. Likewise, some terrestrial biota are capable of withstanding temporary inundation by behavioral, morphological, and physiological adaptations [118]. Inundation and summer groundwater depth were key factors governing the distribution and abundance patterns of vegetation, gastropods, and ground beetles in the floodplain grasslands of the Elbe River [119]. Following an extreme flood with a 168-y recurrence interval, vegetation and gastropods still reflected long-term inundation and groundwater depth patterns because they possessed resistance adaptations. In contrast, carabid beetles were less equipped to resist the extreme flood and were decimated [120]. Another strategy (“resilience”) employed by biota that do not withstand drying or inundation locally is to recolonize from elsewhere. Depending upon life stage and mobility traits, dispersal to a reach following drying or resumption of flow can be active (e.g., flying, crawling, swimming) and/or passive (e.g., anemochory, hydrochory, zoochory). Biota use cues to trigger passive and active dispersal [121,122], but only actively dispersing biota can use cues to terminate dispersal [123,124]. While dispersal can stabilize biotic assemblages to reflect long-term hydrologic conditions, dispersal also enables connectivity across a mosaic of habitats with varying flow duration for different life stages [125]. By expanding the habitat template that biota can inhabit, dispersal between reaches of varying flow duration can enhance growth, survival, and reproduction [126,127] but may limit the use of some biota as flow duration indicators, particularly when reaches with different flow duration classes are in close proximity.

Linkages among morphological, physiological, behavioral, and/or life history traits have been identified as suites or syndromes for dispersal, pace-of-life, or stress under different environmental conditions and ecological contexts [128–130]. Recognizing the suites of traits commonly associated with flow duration classes is useful for targeting potential indicators for SDAMs. Lifespan, time to reach maturity, mobility, and consistency of environmental requirements for life functions are traits of biotic indicators that are likely to discriminate among flow duration classes [131,132]. For example, while there are a few freshwater bivalves that are able to aestivate within sediments to withstand drying (e.g., *Uniomerus tetralasmus*, *Toxolasma paulus*, *Musculium partumeium*), most species are poorly adapted to long periods of drying because they have limited mobility and require flowing water for many life functions (e.g., respiration, filter-feeding, reproduction) [133]. Historically, freshwater unionids were referred to as “naiads” after the Greek goddesses of flowing waters who would expire if their water body dried up. Diametrically, lichens are terrestrial examples of sedentary, slow growing biota, many species of which are not adapted to extended inundation or flowing water. The bulk of lichen diversity associated with freshwater environments is along marginal zones with varying frequencies of inundation [134,135] with few aquatic species requiring permanent inundation [136,137].

## 4. Conceptual Framework for Data-Driven Components of SDAMs

**Figure 3.**
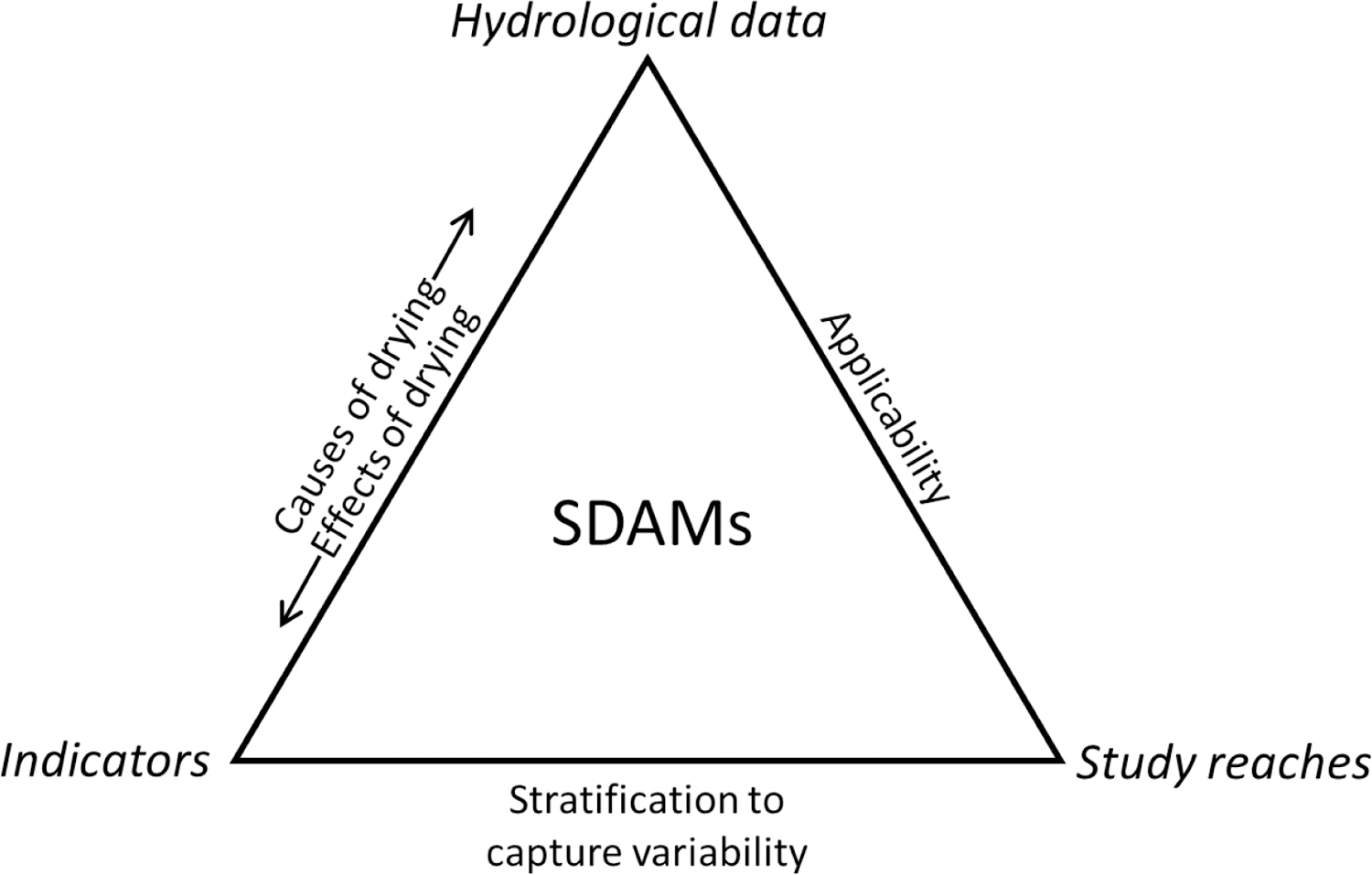
Key components of streamflow duration assessment methods (SDAMs) and their inter-relationships. The arrows moving from indicators to hydrologic data represent indicators that control stream flow duration, whereas the arrow moving from hydrologic data to indicators represent indicators that respond to stream flow duration. Applicability is the selection of study reaches reflecting the intended range of streamflow duration and hydrologic conditions for the developed SDAM. Stratification or regionalization captures variability to improve certainty in the SDAM classification.

The relationship among indicators, study reaches, and hydrologic data represents the conceptual framework upon which SDAMs are built (Figure 3). The relationship between direct hydrologic data and indicators reflects the cause and effect relationships between stream drying and physical and biological environmental variables. Study reaches need to reflect the range of streamflow duration and hydrologic conditions as quantified by hydrologic data observed at the reach-to catchment-scale. Regionalization and stratification of SDAM study reaches through site selection may account for the natural and anthropogenic spatial variability (e.g., climate, biogeography, land use) to ensure environmental indicators can consistently and accurately distinguish flow duration classes throughout space and time within a targeted geographic area.

### 4.1. Indicators

As previously described, indicators can be physical or biological and either responses to or controls of flow duration. Although SDAMs may include control variables, the purpose of SDAMs is to predict flow duration not to provide causal insight for why flow at some reaches has longer or shorter durations than at other reaches. Meteorology, geology, and land cover controls describe the balance between the supplied water and the extent and permeability of the streambed. The strength of an aquatic indicator’s signal will tend to increase with longer flow duration, whereas the strength of a terrestrial indicator’s signal will tend to decrease. Consequently, a standardized protocol that uses a combination of aquatic (e.g., macroinvertebrate life history traits) and terrestrial (e.g., presence of woody vegetation) indicators of flow duration should capture the full flow duration spectrum within a region. The discriminatory ability of indicators will vary spatially and temporally as their relationships to flow duration are linked to other environmental gradients; therefore, indicators reflecting flow duration over longer periods of time (e.g., years) are favored over those that reflect more transient flow conditions. Resistance and resilience mechanisms possessed by biota enhance the representation of flow duration protracted over many years as opposed to the recent hydrologic conditions. Biological traits reflect the adaptations possessed by indicators to cope with varying degrees of environmental fluctuations.

### 4.2. Study Reaches

Study reaches for SDAM development are selected to capture variability of the primary spatial and temporal controls on, and responses to, streamflow duration. Assessing indicators at a sufficient number of reaches to adequately represent key gradients will help produce a robust method for the target region. There is no scientific reason to stratify reaches within a study region by control indicators versus response indicators of flow duration; however, most response indicators of flow duration are measured at the reach scale (Table 1), and it would be impractical to stratify study sites across large regions by many response indicators (e.g., presence of macroinvertebrates, amphibians, substrate sorting). If the target study region is small enough, and datasets pertaining to relevant reach-scale indicators exist at the appropriate spatial scale, reaches could be stratified based on reach-scale response indicators, such as the presence or absence of an indicator species. At a minimum, the study design should consider stratifying reaches of varying flow duration across the three dominant controls of intermittency: meteorology, geology, and land cover [17]. Existing frameworks, such as hydrologic landscapes [138], were designed to characterize similar hydrologic settings across the U.S. and can be used to stratify study reaches across controls of stream intermittency [24]. Similarly, the North American ecoregion framework was designed for studies that aim to analyze subjects that reflect the intersections of multiple abiotic and biotic controls such as stream flow duration [139]. Frameworks that aim to capture variability in flow regimes [140,141] may also be useful, even if not explicitly addressing flow duration, to identify areas or seasons with distinct flow regimes, such as snowmelt versus groundwater dominated reaches or catchments. While existing hydrologic and ecoregion frameworks can be used to guide reach selection to ensure adequate representation of flow conditions within a region, we do not recommend using existing frameworks to establish a priori flow duration classes of study reaches due to the large spatial scale at which most frameworks are developed.

### 4.3. Hydrological Data

Direct hydrologic data (e.g., long-term flow records) that can discriminate among streamflow duration classes are critical for SDAM development. Hydrologic data are described as direct because they need to describe the actual hydrological conditions at a reach and be independent from the environmental indicators. For instance, visual observations of sediment sorting or riparian vegetation from remotely sensed images or field visits should not be used to determine flow duration of study reaches to avoid circularity in the method development process, which could lead to model bias.

Direct hydrologic data are broadly divided based on measurement frequency into (1) daily or more frequent and (2) less frequent than daily (e.g., monthly, seasonally). Daily or more frequent data are typically collected using deployed devices with data storage capabilities and referred to as continuous data, whereas less frequently collected data are referred to as discrete data. Continuous hydrologic data include the measuring of stage, velocity, water temperature, electrical resistance, and time-lapse imagery [142–144]. Less frequent observations or discrete data are typically collected using field observations (e.g., in-person, field cameras, landowner interviews [87,145,146] or remotely sensed observations (e.g., aerial photos, satellite images; [7,147,148] of flow status. Although continuous, long-term data are generally preferred for flow classification, such data collection is limited, and typically biased towards streams with longer flow duration. To ensure the adequate representation of nonperennial reaches in SDAM development, discrete or short-term hydrologic data may need to be considered.

Direct hydrologic data are used to calculate flow metrics which summarize magnitude, frequency, duration, timing, and rate of change characteristics of streamflow regimes [149]. Hydrologic classification can be driven by the data to identify groupings of flow regimes of which flow duration may be one aspect [35,150,151]. Alternatively, hydrologic classification can be driven by narrative definitions that are translated with particular metrics and associated thresholds to classify reaches based on their hydrologic data. The latter is a predominant approach used for flow duration classification with the percentage of time with zero flow being most common (Table 2). When a continuous and complete hydrologic record is available, metrics can be used to repeatably and unambiguously translate hydrologic data into flow duration classes.

In general, the certainty of flow classification from direct hydrologic data increases with the duration, completeness, frequency, recentness, and spatial resolution of the record. Analyzing data from longer records will ensure that flow classification is accurately determined within and across years, while accounting for natural temporal variability. Additionally, longer records may capture human-caused or natural changes on the landscape that have influenced the flow classification over time. If such changes have occurred, the recent hydrology data are preferred for SDAM development to reflect the contemporary flow duration. Higher frequency data also ensure that flow classification is accurately determined, as changes may occur at relatively short time intervals (e.g., on a diurnal, weekly, or monthly basis) that influence inferences about flow duration. For example, some streams dry at night when glacial or snowmelt is low, which would likely not be captured by visual observations during the day [152]. Additionally, continuous data are important for classifying streams that do not flow predictably, given uncertainty about when to conduct field observations during the year. Finally, higher spatial resolution data can increase the accuracy of flow classifications. For instance, aerial imagery may not capture very low flows or obscured flows (e.g., below tree canopies or beneath boulders) that would be evident based on field observations. Direct observations, time-lapse imagery, and in some situations, aerial imagery can capture hydrologic conditions (e.g., dry channel, isolated pools, interstitial flow) [9,10] more resolutely than what is typically captured by gauging stations, temperature, or electrical resistance dataloggers. Careful deployment and/or additional information may be needed to confirm that some continuous datasets can differentiate standing from flowing water or frozen from dry conditions [11]. Parallel use of discrete and continuous datasets and/or the deployment of longitudinal arrays of recording devices can inform flow duration and surface flow connectivity over larger areas [3,54,153].

Direct hydrologic data may be insufficient as a sole source of information to determine flow duration classification. For instance, streamflow data collected during a year of high or low precipitation or temperature may not be representative of a reach’s flow classification under normal conditions. Streamflow data can be supplemented by jointly considering climatic and/or groundwater data to inform classifications with a long-term lens [54,154]. Supplemental datasets can be used to identify portions of the hydrologic dataset that represent extreme or supraseasonal flows [155] and thereby guide flow duration classification, site selection, and/or data interpretation.

## 5. Operational Framework for SDAM Development

**Figure 4.**
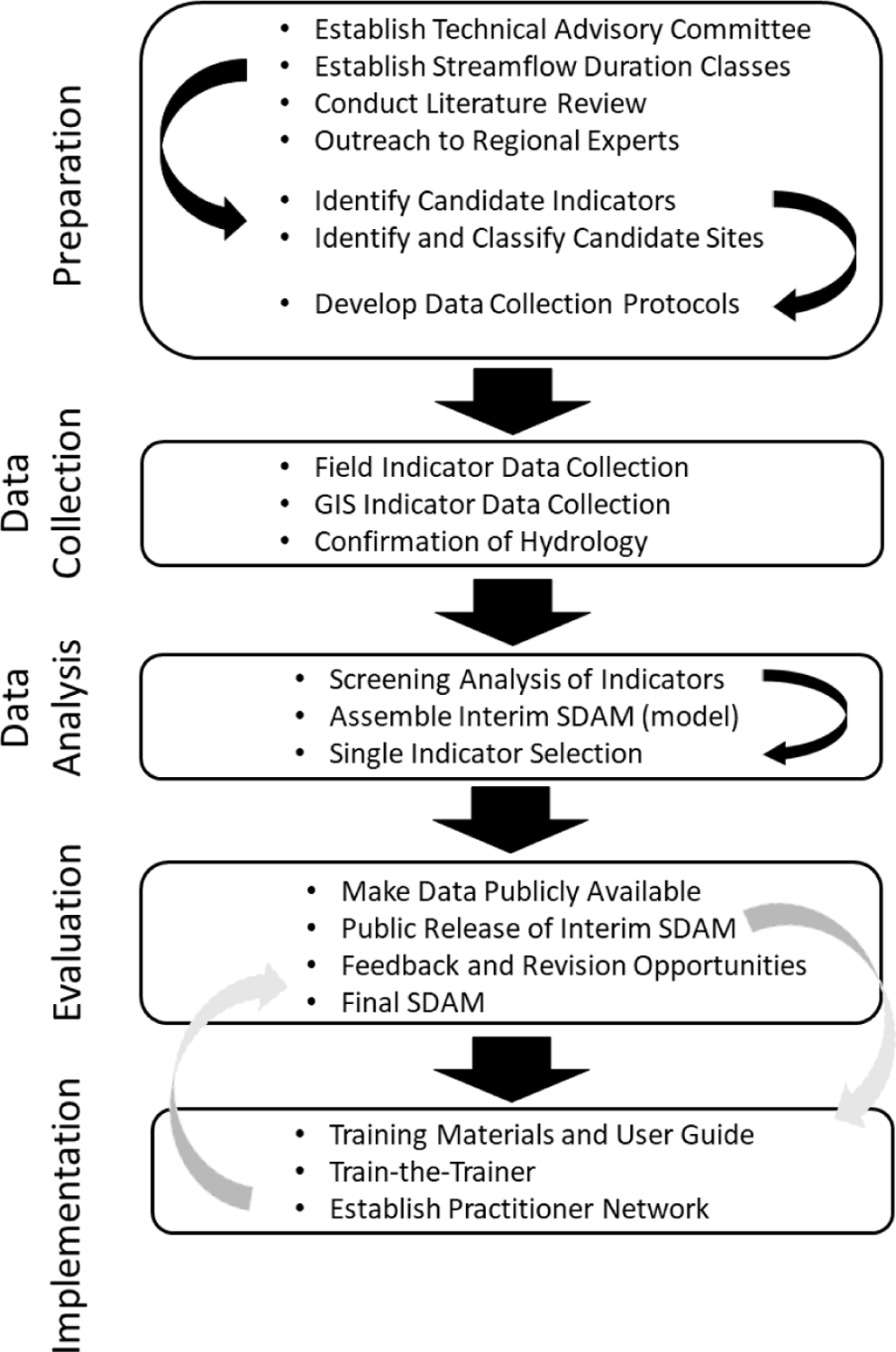
Flow chart showing operational framework for SDAM development. Small black arrows indicate stepwise actions within a process step. The greyarrows denote that implementation actions are iterative, ideally supporting public release of both an interim and final SDAM.

We provide an operational framework for the development of rapid, accurate, robust, and consistent regional SDAMs (Figure 4), outlining the process steps needed to successfully integrate the data-driven components of SDAMs into usable methods: preparation, data collection, data analysis, evaluation, and implementation. These process steps outline how to include stakeholders and end-users throughout method development to ensure users have trust in the SDAM development process and that SDAMs are used consistently and appropriately within a region.

### 5.1. Preparation

#### 5.1.1. Establish Technical Advisory Committee

A technical advisory committee (TAC) with local and regional knowledge makes important contributions [156] to several of the steps outlined (Figure 4) for the development of regionally applicable SDAMs. TACs not only provide technical vetting of the science, they can also improve the adoption of tools and other work products by the organizations they represent. A TAC would ideally include scientific staff from state, tribal, federal, and local agencies and organizations working in related water resource management, land management, and monitoring programs likely to use the SDAM. At the preparation stage, prior to field validation, contributions include identifying regionally appropriate indicators of streamflow duration either formally or informally used, existing sources of direct hydrologic data, possible study reaches, and area-specific considerations such as unusual or challenging hydrologic conditions or unique management needs. Additionally, TAC members increase interdisciplinary connections to other area practitioners and organizations—public, private, and nonprofit—who may provide similarly relevant information useful to study design and implementation.

Following data collection and data analysis, an effective TAC also provides local input and review contributing to the development of the initial, interim SDAM which helps to assure the applicability and usability of the interim SDAM and engages local stakeholders for successful interim method release and use. A TAC continues to serve as a local forum for outreach during the period of local use and evaluation after the release of an interim method, which supports consideration of local feedback in producing a final revised method. Ideally, TAC members form the core of a regional practitioner network which supports method implementation.

#### 5.1.2. Identify Streamflow Duration Classes

SDAMs are typically developed to support the implementation of monitoring, regulatory, assessment, or other water-related programs that benefit from an understanding of flow duration. Therefore, identifying flow duration classes for SDAM development should be informed by the water resource management needs of targeted end-users. One challenge in developing an SDAM for application across a region is that potential end-users may have differing management needs and may define flow duration classes differently. For example, while riparian buffer rules within a region may require definitions of perennial, intermittent, and ephemeral reaches [157,158], water quality standards programs may only require definitions of perennial versus intermittent reaches. Similarly, some programs may distinguish between different types of intermittent streams. The Texas water quality standards program distinguishes intermittent streams from intermittent streams with perennial pools [159], and Ohio distinguishes between warm-water streams with intermittent flow and cold-water streams with interrupted flow [160]. It is also important to consider how flow class definitions will be implemented, as data to inform assessments are often limited to what can be observed at a reach in a single visit. However, it may not be possible to develop a rapid field assessment method to distinguish between types of nonperennial streams without first having a hydrologic record that distinguishes between those classes. Regardless of the number of classes defined for the development of an SDAM, if the described data-driven elements are integrated into the development process, resulting SDAMs will support more objective, accurate, and consistent reach-based classification of streamflow duration.

#### 5.1.3. Conduct Literature Review and Outreach to Local Experts

A two-pronged approach to identifying potential study reaches having direct hydrologic data available and identifying regionally useful indicators of flow duration includes conducting a literature review and consultation with local practitioners. Data quality objectives for literature review data should emphasize the quality of the data sources used in the review, including the utility of data sources to identify flow duration and potential indicators, and support evaluation of indicator applicability in the study region. Possible data sources include established assessment methods, field protocols, scientific reports, grey literature, and peer reviewed publications that seek to classify streams by flow duration and/or evaluate potential indicators of flow duration in the study region. Several assessment factors may be used to evaluate possible data sources, including applicability, utility, soundness, clarity, completeness, and documentation of variability or uncertainty. The TAC may be a useful resource for identifying contacts and conducting outreach with local experts, such as field scientists, water resource managers, land managers, park rangers, citizen scientists, and others familiar with local catchment hydrology or managing or generating hydrologic data. A comprehensive literature review, along with local expert input, informs the identification of potential study reaches and results in a list of potential indicators of flow duration supporting the development of a regional SDAM, which will then be narrowed to a list of final indicators during model development.

#### 5.1.4. Identify Potential Indicators

Potential indicators of flow duration can be characterized by type (hydrological, geomorphological, biological) and endpoints used to assess the indicator. Indicators may be qualitative, such as using ordinal scoring of physical and biological characteristics based on the degree to which that characteristic is observable along the reach, or result in discrete or continuous quantitative data, such as macroinvertebrate abundance or percent slope of the reach. Some may be indicators already in use, or those showing potential to discriminate flow duration classes based on literature review and consultation with local practitioners. Potential indicators should be evaluated for study inclusion based on consistency, repeatability, defensibility, rapidness, objectivity, robustness, and practicality.

There are often tradeoffs in applying the criteria that are used to evaluate potential indicators for inclusion in SDAM development studies. For instance, an indicator that can be assessed rapidly may lack objectivity and repeatability, having been modified from a more quantitative, resource intensive measure assessing characteristics of streamflow duration. One example is the sinuosity index, a quantitative measure of reach sinuosity requiring calculation of the ratio of channel thalweg length to valley length [161]. The measurement of this index in the field requires the use of a surveying level and compass, but this time-intensive method has been replaced with a visual estimation of the number of bends within a stream reach [160] or ordinal scoring using narrative descriptions of the sinuosity index [158], which trades accuracy (and potentially repeatability) for rapidity of measurement. Such tradeoffs can be improved by providing guidelines for establishing standardized assessment reaches and consistent training of end-users [162]. Evaluating indicators for inclusion or exclusion in an SDAM development study, and ultimately for a final SDAM method, is often an iterative process that evolves as the limitations of indicators are revealed during data collection, analysis, and method evaluation.

#### 5.1.5. Develop Data Collection Protocols

Following the evaluation of potential indicators for inclusion in the study, a protocol providing detailed instructions on preparing for field data collection and assessing selected indicators can be developed. Such a protocol is critical to ensure consistent data collection, quality of data collected, and efficiency in the field. Importantly, a data collection protocol for the field study also serves as a precursor to the development of a user guide supporting method implementation.

An important aspect of a stream assessment protocol is describing the boundaries of the assessment reach in a way that can be consistently applied by multiple practitioners. Flow characteristics often vary along the length of a stream, resulting in transitions in flow duration and indicators. Because flow duration can vary over short distances, the delineation of standard assessment reaches at study sites needs to be clearly described. For instance, in a study of 264 stream reaches supporting the development of an SDAM for the Pacific Northwest [24], standard reaches are equivalent to 35–40 channel widths of the stream [163], and a minimum assessment reach length of 30 m is set for narrow streams. Reach length is measured along the thalweg, and if the target reach is near a culvert or road crossing, the assessment reach begins at a minimum of 10 m from the feature. If a reach is not uniform, two or more representative reach assessments are recommended to fully describe the changes along the reach; these guidelines for identifying standard assessment reaches are maintained in the final, implemented SDAM [164]. In the Fairfax County, VA, protocol for identifying perennial streams [165], standard assessment reaches are described as at least 61 m (200 ft) and having similar physical characteristics potentially bounded by an upstream and downstream tributary, grade control, other physical feature, or an obvious change in channel characteristics.

Several additional elements should be covered in a comprehensive data collection protocol to ensure that data quality and efficiency objectives are met. Especially given that some measured indicators may be ordinally scored based on visual estimates (e.g., presence, absence, extent), which are subjective measurements including subjective interpretation [166], a precise written description of how to assess each indicator is essential. It should include specific instructions for instrumentation, photodocumentation, and the collection, treatment, and identification of biological organisms, as appropriate. Common circumstances where data collection protocols may need to be adjusted are ideally anticipated and described to support consistent decision-making in the field. Finally, a quick-reference field work of operations, complementing electronic or hard copy field forms, promotes a streamlined approach to data collection.

#### 5.1.6. Identify and Classify Study Reaches

We describe both a resource-intensive and a resource-limited approach to identify reaches for SDAM development. Both approaches require reaches that are representative of the range of hydrologic conditions within a region. For the resource-intensive approach, direct hydrologic data to inform a priori flow duration classifications is preferable, but not required, because reaches are either instrumented or regularly monitored to document flow at the reach-scale. Previous monitoring efforts (e.g., instrumentation or reach visits) and knowledge of controls on flow duration within a region can help to characterize hydrological conditions at the reach scale and a priori flow duration classifications. For example, spatial data and models combined with knowledge of underlying geology, precipitation patterns, and anthropogenic influences can inform the stratification of reaches within a region. Once reaches are identified based on previous monitoring data or regional controls on flow duration, reaches are instrumented to monitor the presence or absence of water over time (e.g., with cameras, temperature or conductivity data loggers). If instrumentation is not feasible, reaches may be visited multiple times during wet and dry seasons; in certain regions, visits may be timed to provide the best information about flow duration (e.g., visiting streams at the peak of the dry season in Mediterranean California) [167]. Knowledge of predictable patterns in local hydrology and other anecdotal evidence provided by local experts (i.e., best professional judgement) may inform reach selection through a resource-intensive approach because hydrology will also be monitored throughout SDAM development. However, when considering best professional judgment, it is important to focus on hydrologic observations as opposed to indirect indicators of flow duration (e.g., vegetative cover, macroinvertebrate community structure) to avoid confirmation bias when choosing indicators for inclusion in the final SDAM. For example, if a local expert recommends an ephemeral reach because surface water is rarely observed during frequent and well-timed visits, this reach would be a good candidate for the study. In contrast, if an expert recommends an ephemeral reach because it lacks typical riparian vegetation, including this reach would introduce circular reasoning when defining a priori flow duration classes and potential model bias if riparian vegetation is included as an indicator of flow duration during data analysis. Reaches selected through a resource-limited approach must use direct hydrological data from previous monitoring efforts to inform a priori streamflow classifications. Spatial data and knowledge of controls on flow duration within a region may help to stratify study reaches by hydrological condition, but such knowledge must be combined with direct hydrological observations at the reach scale for reach selection. If possible, resource-limited study reach selection should not rely on best professional judgement alone to make a priori flow duration classifications.

There are advantages and disadvantages to both resource-intensive and resource-limited reach selection approaches. As described, the resource-intensive approach requires more time to monitor sites over multiple wet and dry seasons and has greater costs associated with instrumentation and site visits compared to the resource-limited approach. However, due to the limited availability of high-frequency, long-term hydrological monitoring on nonperennial streams [11,168], the resource-limited approach is constrained in its ability to adequately identify reaches that represent a range of hydrologic conditions, as nonperennial streams will likely be underrepresented. If a moderate level of resources is available, the two approaches could be combined. A subset of previously visited reaches where flow duration is inferred through best professional judgement could be instrumented, and visited multiple times throughout SDAM development, while reaches with a long-term and recent continuous hydrologic record do not need instrumentation.

### 5.2. Data Collection

#### 5.2.1. Collect Field Indicator Data

To capture temporal variability of measured indicators, we recommend sampling all study reaches during times when intermittent streams are expected to be both wet and dry. Visiting perennial streams when nonperennial streams are expected to be dry can help to confirm that perennial streams do not dry, even during dry seasons. Visiting nonperennial streams when they are expected to be dry helps to confirm that they are not perennial and visiting intermittent streams when they are expected to be flowing can help confirm that they are not ephemeral. Not all regions have strong seasonal patterns in drying (see [40]), in which case assessing reaches multiple times per year can help capture seasonal variation in flow duration indicators. Planning for reach visits should also consider prolonged stressors such as drought and discrete disturbances such as floods and channel-modifying activities (e.g., channel armoring, bank stabilization, revegetation) that can temporarily obscure observations of indicators that normally help discriminate among flow duration classes. Recent channel modifications or large flood events can obscure geomorphic indicators such as soil texture and recent alluvial deposits. Some existing methods recommend not assessing reaches within 48–72 h of significant rainfall to ensure that indicators are not observed directly after disturbance [158,169,170]. Similarly, abnormally long periods without precipitation can obscure biological indicators such as the presence of hydrophytic plants and macroinvertebrate community structure. The New Mexico Hydrology protocol strongly recommends not assessing reaches during a drought period and provides a Standardized Precipitation Index threshold to indicate severe to extreme drought conditions [169]. We recommend assessing reaches under normal climatic conditions and after an appropriate recovery period from major anthropogenic disturbances.

#### 5.2.2. Collect GIS Indicator Data

Most GIS derived indicators of flow duration will reflect controls on flow duration at the catchment or larger spatial scales. Existing regional and national data layers, for example StreamCat [171] and national coverages within the database (e.g., National Land Cover Dataset, Parameter-elevation Regressions on Independent Slopes Model (PRISM)) can provide spatial data that reflect many of the geological, meteorological, and land cover controls on flow duration. Within homogenous ecoregions or hydrologic landscapes, catchment area can correlate with flow duration [36]. Catchment area thresholds between intermittent and perennial headwater streams were derived in the northeast U.S. [37,170], as well as between ephemeral and intermittent streams in western U.S. [45]. As with most landscape scale controls on flow duration, such as catchment area and precipitation, thresholds between flow duration classes are rarely universal within and among regions. For example, Olson and Brouillette [170] constrained catchment area thresholds between intermittent and perennial streams to < 12.9 km^2^, and Hedman and Osterkamp [45] excluded streams of the arid southwest from catchment area and flow duration relationships in the western U.S. GIS control metrics can be used throughout SDAM method development to inform reach selection as well as during model development to account for landscape-level differences in indicator effectiveness.

### 5.3. Data Analysis

#### 5.3.1. Screening Analysis of Indicators

The purpose of screening analysis is to narrow the list of measured indicators to the candidate indicators for an SDAM. Screening indicators first involves reviewing data for outliers, independence, and other underlying statistical assumptions [172]. Many steps used for developing indices for biological condition assessment [173,174] are helpful for screening SDAM indicators (particularly those indicators derived from biological assemblage data). If data collection involved collecting raw continuous data (e.g., abundance or % cover of species assemblages), an initial step may be needed to calculate indicators (i.e., metric calculation) that summarize specific aspects or subsets of the raw field data (e.g., traits, taxonomic groups, diversity metrics) and/or make the data comparable across sampling units (e.g., relative abundance) if qualitative or semiquantitative sampling was used.

There are several simple screening tests that can identify which measured indicators have desirable properties, such as good discrimination ability or high precision. Range tests eliminate indicators with small ranges that are not useful in discriminating between flow duration classes. Reproducibility tests identify indicators whose values are consistently associated with flow duration classes. A common way to evaluate reproducibility is the ratio of the variance of indicator values from visits among all reaches (“signal”) to the variance of indicator values from visits to the same reach (“noise”). Higher signal:noise ratios indicate higher reproducibility of the indicator. Redundancy among indicators describes whether different indicators provide very similar information or have collinearity regarding flow duration. Redundancy can be evaluated using Pearson or rank correlation coefficients (e.g., strongly correlated r > |0.71|) or variance inflation factor [172]. In most situations, redundancy or collinearity among candidate indicators will help to detect the effect of individual indicators in the SDAM model but not necessarily the overall model’s ability to predict SDAM classes. Responsiveness (i.e., the ability of an indicator to change along flow duration gradients) is arguably the most important property of SDAM indicators. Measures of responsiveness (e.g., *t*-statistics from comparing mean indicators in two classes of streams) can provide useful criteria for screening indicators, as they describe whether indicators in isolation can differentiate flow duration classes. Indicators should reflect an interpretable and predictable relationship with flow duration. This, however, does not necessarily signify a clear one-to-one or linear relationship between the indicator and flow duration. Indicators can still be judicious in combination with other measures to accurately predict flow duration class. Visualizing the indicator data using boxplots or dotplots are simple ways to evaluate responsiveness. Statistical methods that test for substantial differences among indicators scores, such as *t*-tests [174] and nonparametric Mann–Whitney *U*-tests [175], can help to identify the most responsive study indicators. If complete biological community data are available, indicator species analysis [176] or similarity percentage [177] approaches can identify taxa that are most responsible for assemblage difference at reaches that differ by flow duration. It is important to keep in mind that some indicators may be better at distinguishing between perennial and ephemeral reaches and that ability does not necessarily reflect how well an indicator may discriminate those reaches from intermittent reaches.

The next screening step is the evaluation of the remaining indicators to identify the candidate indicators. Discriminant analysis is one approach to compare the ability of different combinations of indicators to discriminate among flow duration classes and screening indicators [36]. Machine learning approaches such as random forest [178] and conditional inference trees [179] are nonparametric classifications that use ensemble learning (“bagging” or bootstrap aggregation) across many decision trees or Classification and Regression Trees (CARTs) [180] to identify the most important indicators in classifying flow duration. Datasets are randomly split into training and validation sets, and variable importance is measured using the Gini Index or mean decrease in classification error. Fritz et al. [181] used random forest to identify which indicators of an existing SDAM were most important in differentiating among streamflow duration classes in South Carolina. Random forest was applied as a screening step for indicators representing macroinvertebrate traits to predict hydrologic state (flowing vs. disconnected pools) at the time of sampling from seven Mediterranean streams [182]. One advantage of such nonparametric machine learning methods is that the only underlying assumption is that sampling is representative of conditions for which the SDAM could be subsequently used, making reach selection a particularly important step in SDAM development.

#### 5.3.2. Assemble Interim SDAM

The assembly of interim SDAM models is the evaluation of combinations of candidate indicators that most accurately predict the flow duration class membership of stream reaches. There are three formats that have been produced for applying SDAM models: multimetric indices, linear equations, and decision trees. The format of SDAMs in large part reflects the data analysis approach used to arrive at the best combination of indicators. Multimetric indices are the most common format that has been used in the U.S.; examples include the North Carolina Methodology for Identification of Intermittent and Perennial Streams and Their Origins, Version 4.11 [158] and several derivative indices such as the New Mexico Hydrology Protocol [169], Tennessee Hydrological Determination Guidance, Version 1.4 [183], and Fairfax County Perennial Stream Field Identification Protocol [165]. The assembly of some multimetric SDAM indices are modeled after early biotic integrity indices (IBIs) [184], which at the time were revolutionary advances in stream monitoring, but indicator selection was largely based on hypothesized relationships and expert judgement. Multimetric SDAM indices use ordinal scoring of physical and biological indicators based on the degree (e.g., strong, moderate, weak, and absent) to which each indicator is observable along the reach. The ordinal degrees to which indicators are observable are weighted numerically (e.g., 3, 2, 1, 0 or 1.5, 1, 0.5, 0) to reflect the potential utility of the indicator for discriminating among flow permanence classes. The numerical values are summed across the indicators, and this total index score is used to assign a flow permanence class based on thresholds between classes [185]. A multimetric index was developed based on the plant, mollusk, and carabid beetle indicator species to predict classes for the inundation duration per year and mean depth of groundwater during the growing season of a German grassed floodplain [119]. Each indicator species was assigned an indicator value as the abundance-weighted mean value for weeks of inundation per year and for growing season groundwater depth in meters from a subset of the samples. The multimetric index score was derived as the weighted mean indicator value of all recorded indicator species at a reach. Proponents of the multimetric approach consider redundancy among metrics to afford robustness to errors or variability that may affect a single metric. However, metric redundancy may compound error or inflate signals from indicators that are not singularly responsive to flow duration.

SDAMs that use linear equations have primarily derived from logistic regression for predicting the probability that a reach does (true probabilities > 0.5) or does not (false probabilities < 0.5) belong to a flow duration class. Logistic regression equations have been developed for Massachusetts [37,186], Vermont [170], and the Triassic Basins Ecoregion in North Carolina [187] using catchment-scale indicators (as opposed to field-based measures collected from reaches). The best equation was selected using a Hosmer and Lemeshow goodness-of-fit test, receiver-operating-characteristic curves, and regression diagnostics (e.g., Akaike’s Information Criterion, Kendall’s Tau-a, McFadden’s pseudo R^2^). The flow duration class was determined by calculating probabilities using the best logistic equation, and associated uncertainty could be described with upper and lower confidence limits of the predicted probabilities. The probability cutpoint is the optimized threshold for correctly assigning flow duration class to reaches. Logistic regression was used to predict the probability of plant species occurrence in relation to the inundation duration along the Fremont River in Utah [188]. Species probabilities were used as weights for a wetland prevalence index developed for predicting the inundation duration class (aquatic, transitional, and upland). Least-square regression equations using catchment-scale indicators have been used to differentiate flow duration classes in Idaho, where the annual minimum flow is a surrogate for flow duration [38]. Related to SDAMs, logistic models with catchment-scale indicators have also been used to predict the probability of stream reaches being wet or dry at different parts of the year [189]. Straka et al. [175] developed an SDAM index (i.e., Biodrought index) that discriminated among three flow duration classes (perennial, near-perennial, and intermittent) in minimally disturbed streams of the Czech Republic. The index was assembled using discriminant analysis to identify the best linear combinations of macroinvertebrate indicators from spring and autumn samplings. The authors then adjusted the equation so that the resulting index score was centered (i.e., zero) on the flow duration gradient across study reaches such that strongly positive scores reflected a high probability of being perennial, whereas strongly negative scores reflected a high probability of being intermittent [175].

Decision trees are an increasingly common classification approach that optimizes prediction over understanding. Earlier we described CART and random forest as decision tree approaches for screening indicators. These same approaches can be used to assemble interim SDAMs using sets of candidate indicators. Decision trees are versatile in the data types that can be used; indicators are not evaluated in a linear manner and do not have the underlying assumptions of other approaches. One potential disadvantage of decision tree approaches is that they can be unstable, where small changes in the data used to inform the model can result in major structural changes in the resulting tree. The drawing structure from many decision trees, as performed using random forest and gradient boosting, helps to address this problem. Selecting representative study reaches for SDAM model assembly is important because CART, gradient-boosted trees, and random forest are data-driven (rather than process-driven) machine learning models which are particularly susceptible to poor performance when extrapolated. Fundamental to decision trees is the identification of optimal splits (thresholds) in indicators that discriminate between subsets of classes. This helps to simplify the eventual application of decision tree SDAMs for indicators that were originally more meticulously quantified as continuous measures into one of two categories—above and below the split—for more rapid field application. The optimum decision tree, however, can use the same indicator more than once in the tree and have different splits. The Pacific Northwest method is an example of an SDAM that initially used random forest as an indicator screening tool then used CART to assemble the interim SDAM model from the set of candidate field indicators, and the tree has two different splits for channel slope [24]. Cid et al. [182] used a similar approach to develop a decision tree using macroinvertebrate indicators for predicting the aquatic state (flowing or dry) of Mediterranean stream reaches. Combinations of physical habitat and amphibian indicators were used in a CART model to predict flow duration classes for reaches in forested headwater streams [27]. By using a cross-validation process to remove the least important predictors and subsequently testing the prediction power of reduced models, parsimonious decision trees for predicting flow duration can be developed. Model fit is evaluated using regression diagnostics, such as percent of variation explained (pseudo-R^2^), observed vs. predicted class (false positives, false negatives), Cohen’s kappa, residual distribution, and root mean square error. Random forest-based SDAM models using control indicators to predict flow duration class in river networks have been developed for the Upper Colorado River basin [39], northern Rocky Mountains [84], France [190], and northern Spain [191]. Another related approach that has used machine learning to classify flow duration using control indicators has predicted continuous probabilities of year-round flow rather than discrete flow classes [35].

#### 5.3.3. Single Indicator Selection

An additional strategy that some existing SDAMs have incorporated is the use of single indicators. Single indicators are those indicators that provide strong discriminating information about a reach alone or in conjunction with the SDAM model prediction. For instance, the presence of fish (except *Gambusia*) and particular amphibians and reptiles that require sustained water presence are used in the Pacific Northwest SDAM as single indicators to indicate that reaches are at least intermittent. The absence of single indicators, however, is not indicative of flow duration class because they are commonly not found in intermittent and perennial reaches, but when they are present, they are accurate indicators of those flow classes [24]. Similarly, NCDWQ [158] also uses single biological indicators in conjunction with a multimetric SDAM, but their presence indicates that reaches are perennial. Single indicators may be designated early in the indicator selection process step when the existing literature, previous studies, or expert consensus strongly supports their utility for discriminating flow duration. Because of their limited distribution across study reaches, some indicators screened out during data exploration may be suitable to investigate as single indicators. Such indicators should be highly accurate in discriminating flow classes based on their presence or absence, but not necessarily both conditions. Single indicators enhance the rapidity of SDAMs by providing an accurate classification using only a single indicator, rather than collecting data for an SDAM model in its entirety to predict flow duration class.

### 5.4. Evaluation

#### 5.4.1. Provide Evaluation and Feedback Opportunity

To ensure usability and applicability, we recommend making an interim SDAM publicly available [156] for a minimum of one year to allow the user community the opportunity to provide feedback on their experiences using the method across seasons. Evaluation and testing of an interim method, including identifying strengths and weaknesses to inform the production of a final method, could range from voluntary use to more formal efforts aimed at specific features of the model. For instance, a more formal approach includes intensification studies [192] in which a state, tribal, or other organization invests resources and leverages study designs to collect data at additional reaches in the region of interest to produce a finer-scale evaluation of an interim SDAM. The latter approach could be especially beneficial if an interim SDAM model was developed for a very large area and would benefit from a finer-scaled distribution of study reaches in hydrologic landscapes not adequately represented in the initial field study.

Additionally, we recommend that the complete, quality-assured dataset from which the interim SDAM model was assembled be made publicly available. This ensures transparency and provides access for parties interested in additional data analysis. This transparency is one of the fundamental open-science principles that advance the adoption of technical products into environmental management decisions, and builds credibility for tools by regulated parties, advocacy groups, and other stakeholders [193]. It also contributes to the growing collection of streamflow duration datasets that may inform larger scale analyses and other lines of inquiry to which the data are relevant, helping to advance the science of streamflow duration assessment to support management needs.

Regardless of the evaluation approach, a mechanism facilitating user feedback on an interim SDAM model, such as agency-hosted outreach and training events, a dedicated e-mail address or website for receiving user comments, specified agency contact(s), or some combination should be implemented. This will encourage timely feedback and build trust and support for the process and product [156].

#### 5.4.2. Produce Final SDAM

Following the period of evaluation and testing, including any intensification studies, by the local user community, direct feedback received should be considered and incorporated, where appropriate, to produce a final SDAM method. A description of how user feedback and analysis is reflected in the final method, and the value of those contributions in producing a user-friendly final SDAM applicable to the region for which it was developed, should be communicated [156] with the public release of the final SDAM.

### 5.5. Implementation

There are several actions that contribute to the efficient implementation of an SDAM and ensure that it is used effectively in informing streamflow classification determinations. To optimize outcomes, the described actions should be iterative, supporting the public release of both an interim and final SDAM.

#### 5.5.1. Prepare User Guide and Training Materials

A user guide provides detailed instructions for collecting data regarding the described indicators of streamflow duration, completing necessary field assessment forms and evaluating the results to distinguish between described classes of flow duration. It helps to ensure consistency, repeatability, and efficiency in SDAM application by practitioners, and the documentation of results. This, in turn, facilitates output review and concurrence, and supports the defensibility of outputs used to inform decision-making.

While a user guide serves as a foundational training document, because training is vital to ensure that science is used in practice [194], we recommend developing a standard training module incorporating training materials and format. This facilitates providing a consistent level of training to end-users and has the added benefit of being easily transferable among trainers. For a field-based SDAM, both classroom instruction (or a distance learning equivalent) and field exercises should be included in the training format.

#### 5.5.2. Deliver Training and Establish Practitioner Network

The delivery requirements of turning science into practice can be considerable [194]. Additionally, while training enables a wider number of end-users to fully appreciate the potential of a method [194], implementing agencies and organizations often lack the staff and resources to deliver direct training to the universe of potential end-users with the desired frequency to sustain a practitioner network.

The Train-The-Trainer model, in which a smaller number of people are trained to disseminate the knowledge as in-house experts for their organization, offers several advantages to mitigate these constraints. It is cost-effective, provides consistency in delivering the training curriculum, helps trainers become subject matter experts, and can be more rapidly deployed than direct training by method developers alone [195]. For these reasons, we recommend using the Train-The-Trainer model to deliver training both internally and externally and to establish and sustain a practitioner network, which in turn supports accurate and consistent method application and implementation.

## 6. Needs to Improve SDAMs and Their Application

There remains a need for increased hydrologic data collection in intermittent and ephemeral streams, because most stream gauges are stationed in perennial streams. While direct hydrologic data are an important component of validating SDAMs and choosing appropriate indicators [24], such data are also critical for other research efforts such as mapping streams, modeling water budgets and water quality, and a variety of other applications [196,197]. Collecting additional continuous data through gauges, loggers, and time-lapse photography should be prioritized for high-confidence streamflow duration classifications that can be used in developing SDAMs. With the evolution of sensor technologies, we anticipate the expanded use of low-cost data loggers that can directly characterize flow duration [198,199]. Such devices reduce resource barriers that prevent the collection of widespread continuous hydrologic data, although data loggers still require routine visits to conduct maintenance, download data, and field-verify recorded data, and there is a lag time between data collection and availability.

Discrete sources of direct hydrologic data also present a strong opportunity for data collection expansion, including data that are obtained through local expertise, indigenous ecological knowledge, or citizen science efforts. Discrete data from these sources may be particularly useful for characterizing small streams which may be difficult to access, difficult to gauge, or obscured from remote images by forest or other cover. For example, citizen science efforts have been successful in some parts of the United States [200–202] and France [18], but such programs should be expanded to better characterize nonperennial streams. We also recognize the need to create standardized approaches for determining streamflow duration class when relying primarily on discrete hydrologic data.

In addition to expanding the spatial coverage of direct hydrologic data, future efforts should also focus on the development of long-term flow records from intermittent and ephemeral streams. Because streamflow duration classes represent the typical regime at a reach over many years, long-term datasets could be used to inform the relevant degree of interannual variability that should be considered when determining typical flow conditions, as well as the appropriate length of record needed to capture that variability. Without the availability of long-term streamflow data, the consideration of climatic data is often necessary to determine streamflow duration class. While there has been useful expansion of accessible weather data that can be geolocated (e.g., PRISM, Snow Telemetry (SNOTEL)), further research is still needed to identify the relevant temporal scales that should be considered when determining whether recent or short-term flow data are representative of typical flow conditions. We see this as an area of research where more interdisciplinary work could be performed between meteorologists and hydrologists to improve streamflow duration classification and inform SDAM development.

Future work should also focus on SDAM indicator development, including identifying the appropriate scope of inference for such indicators across different geographic areas and other natural or human-driven gradients. A promising SDAM frontier is the development of statistical relationships between flow duration and indicators that can be gathered via remote sensing or geographic information system datasets (e.g., [35,191]). The strength of such relationships, as well as data availability, may vary widely across landscapes and should, therefore, be developed on a regional basis. Such information could reduce the need for resource-intensive field studies to classify flow duration. We also advocate for focused research on indicators and SDAMs across different stressor gradients, (e.g., old versus new urban or agricultural development) to confirm applicability and identify controls that could be used for indicator or model stratification.

Importantly, continued work in pursuit of developing functional assessment methods and biomonitoring programs that explicitly consider streamflow duration class will continue to inform our ecological understanding of nonperennial streams, and thus, the development of SDAMs [203–205]. Specifically, the collection of macroinvertebrates from biomonitoring in intermittent and ephemeral streams could be used to improve the identification of regionally-specific indicator taxa for SDAM development. Additionally, increased monitoring efforts in nonperennial streams may also be helpful for identifying other response indicators, determining effective endpoints or thresholds for certain indicators, and improving our understanding of the temporal variability of these indicators in response to flow conditions.

## Figures and Tables

**Table 1.** Examples of response and control indicators of streamflow duration evaluated over spatial and temporal scales. - is not applicable.

Indicator	Response/Control	Spatial Scale	Temporal Scale	References
Bryophytes	Response	Reach	Multi-annual	[20]
Filamentous algal biomass	Response	Subreach to reach	Subannual to annual	[21]
Periphyton pigment concentration	Response	Subreach to reach	Subannual to annual	[22]
Lichens	Response	Subreach	Multi-annual	[23]
Macroinvertebrates presence	Response	Reach	Subannual to multi-annual	[24]
Macroinvertebrate indices	Response	Reach	Subannual to multi-annual	[25]
Riparian vegetation	Response/control	Reach	Multi-annual	[24,26]
Amphibians	Response	Reach	Annual to multi-annual	[27]
Fish	Response	Reach to catchment	Subannual to multi-annual	[28,29]
Sediment sorting	Response	Reach	Subannual to multiannual	[30]
Leaf litter	Response	Reach	Subannual to annual	[31,32]
Wood	Response/control	Reach, Catchment	Multi-annual	[33,34]
Channel slope	Control	Reach	Decadal	[35]
Entrenchment ratio	Control	Reach	Multi-annual	[36]
Catchment area	Control	Catchment	-	[37,38]
Potential evapotranspiration	Control	Catchment	Annual	[39]
Precipitation	Control	Catchment	Daily, annual, decadal	[35,40]
Precipitation- vegetation feedback	Control	Catchment	Annual	[41]
Percent sand and gravel deposits	Control	Catchment	-	[37]
Percent grassland	Control	Catchment	-	[39]
Percent forest	Control	Catchment	-	[35]

**Table 2.** Examples of hydrologic metrics used for flow duration classifications.

Geography	Metric	Flow Classes	Ref.
USA	Percent of year with flow	Intermittent: 25 to <100 Perennial: 100	[44]
Western USA	Percent of year with flow	Ephemeral: <10 Intermittent: 10 to 80 Perennial: >80	[45]
Forested USA	Percent of year with flow	Intermittent and ephemeral: <90 Perennial: 90 to 100	[46]
Great Plains USA	Percent of year with flow	Ephemeral or interrupted: <20 Intermittent: 20 to 80 Perennial: >80	[47]
Huachuca Mountains, Arizona, USA	Percent of year with flow	Ephemeral: 0.01 to 5 Intermittent: 1.5 to 70 Perennial: 100	[3]
USA	Percent of year with flow	Ephemeral: <9 Intermittent: 9 to 99.726 1 zero flow per year: 99.726 to <100 Perennial: 100	[48]
SE Queensland, Australia	Percent of year with flow	Strongly intermittent: <30 Weakly intermittent: 30 to 90 Perennial: >90	[49]
Mediterranean Europe	Mean number of months with flow per year	Episodic-ephemeral: 0 to 7.2 Intermittent-dry: 7.2 to 10.2 Intermittent-pool: 10.2 to 12 Permanent: 10.8 to 12	[10]
	Probability of dry in 6 month dry season	Episodic-ephemeral: 0.25 to 1 Intermittent-dry: 0 to 1 Intermittent pool: 0 to 1 Permanent: 0 to 1	
USA	Percent of year contains surface water	Intermittent: <100 * Perennial: 100	[50]
Burkina Faso	Mean number of months with zero flow in normal and drought years	Strongly ephemeral: 9 Mid ephemeral: 7 Permanent w/ high variability: 12 normal, <12 drought Permanent w/ low variability: 12 but erratic in drought Permanent: 12	[41]
Upper Colorado basin, USA	Mean number of zero flow days yr^−1^	Strongly intermittent: >20 Weakly intermittent: 19 to 1 Perennial: 0	[39]
	Percent of months with zero flow	Strongly intermittent: >5 Weakly intermittent: 5 to 0 Perennial: 0	
Segura basin, Spain	Percent of months with zero flow	Intermittent and ephemeral: 50 to 20 Perennial seasonal: 20 to 0 Perennial stable: 0	[51]
Piedmont region, North Carolina, USA	Months of continuous flow	Intermittent: 3 to 12 Perennial: 12	[52]
Idaho, USA	7Q_2_	Intermittent: <28.32 L/s Perennial: >28.32 L/s	[38]
Okanagan basin, British Columbia, Canada	Minimum daily discharge	Intermittent: 0 L/s Almost intermittent: <5 to 0 L/s Perennial: >5 L/s	[53]
France	Minimum daily discharge over 5 consecutive days	Intermittent: <1 L/s Perennial: ≥1 L/s	[54]
Mediterranean Europe	Mean percent of months with flow	Episodic: 0 to 20 Occasional: 0 to 40 Alternate: 0 to 40 Alternate-stagnant: 0 to 40 Stagnant: 0 to 40 Alternate-fluent: >40 to 90 Fluent-stagnant: >40 to 90 Quasi-perennial: >90 to 99 Perennial: >99	[55]
	Mean percent of months with isolated pools	Episodic: 80 to 100 Occasional: 60 to 100 Alternate: 60 to 100 Alternate-stagnant: 60 to 90 Stagnant: 50 to 100 Alternate-fluent: 0 to <50 Fluent-stagnant: 0 to <60 Quasi-perennial: 0 to 10 Perennial: 0 to <1	
	Mean percent of months dry	Episodic: 80 to 100Occasional: 60 to <80Alternate: 20 to <60Alternate-stagnant: 10 to <60Stagnant: 0 to 10Alternate-fluent: 10 to <60Fluent-stagnant: 0 to 10Quasi-perennial: 0 to 10Perennial: 0 to <1	

* But more than just after rainstorms and at snowmelt.
